# Retraction Note: minimally invasive versus open surgery for acute Achilles tendon rupture: a systematic review of overlapping meta-analyses

**DOI:** 10.1186/s13018-018-0862-6

**Published:** 2018-06-19

**Authors:** Qingbo Li, Chuanying Wang, Yanqing Huo, Zhiwei Jia, Xiqian Wang

**Affiliations:** 1 Department of Orthopaedic Trauma, The Second Hospital of Shandong University, Jinan, People’s Republic of China; 2 Department of Rehabilitation, The Second Affiliated Hospital of Shandong University of Traditional Chinese Medicine, No.1 Jingba Road, Jinan, 250001 People’s Republic of China; 3 Department of Orthopaedics, The 306th Hospital of People’s Liberation Army, Beijing, People’s Republic of China

**Retraction Note:** the authors have retracted this article [1] because of text overlap with a previously published article [2]. All authors agree to this retraction.
